# Synthesis of 3-(phenylazo)-1,2,4-triazoles by a nucleophilic reaction of primary amines with 5-chloro-2,3-diphenyltetrazolium salt via mesoionic 2,3-diphenyltetrazolium-5-aminides

**DOI:** 10.3762/bjoc.5.8

**Published:** 2009-03-02

**Authors:** Shuki Araki, Satoshi Hirose, Yoshikazu Konishi, Masatoshi Nogura, Tsunehisa Hirashita

**Affiliations:** 1 Omohi College, Graduate School of Engineering, Nagoya Institute of Technology, Gokiso-cho, Showa-ku, Nagoya 466-8555, Japan

**Keywords:** formazan, mesoionic compounds, nucleophilic substitution, tetrazolium, 1,2,4-triazole

## Abstract

The reactions of a 5-chloro-2,3-diphenyltetrazolium salt with amines have been examined. In the presence of an inorganic base such as NaHCO_3_, primary and secondary amines undergo a nucleophilic substitution to give the corresponding 5-aminotetrazolium salts. When triethylamine is used as a base, primary amines give 3-phenylazo-1,2,4-triazoles. A plausible dual-path mechanism is proposed for the formation of the triazoles via Type B mesoionic tetrazolium-5-aminides.

## Introduction

Mesoionic compounds can be classified into two families, type A and type B mesoions, according to their electronic arrangements [1–5]. Type B mesoionic compounds are less common compared with type A mesoions. The 5-chloro-1,3-diphenyltetrazolium salt (**1**) has been proved to be a versatile precursor to several type A 1,3-diphenyltetrazolium mesoionic compounds, because the chlorine atom is a good leaving group and easily replaced by a variety of nucleophiles [6–7]. Although the corresponding 5-chloro-2,3-diphenyltetrazolium salt **2** is not yet known, it is expected that **2** is also useful for the synthesis of type B tetrazolium mesoions. In this paper, we disclose the first preparation of this chlorotetrazolium salt **2** and its reactions with amines, which involve the unexpected formation of 3-(phenylazo)-1,2,4-triazole derivatives.

## Results and Discussion

The 5-chloro-2,3-diphenyltetrazolium salt **2** was prepared in an analogous manner to the corresponding 1,3-diphenyl isomer **1** [6–7]. Thus, the treatment of 2,3-diphenyltetrazolium-5-olate with phosphorus oxychloride gave **2** in high yield as stable crystals, after an anion exchange to tetrafluoroborate.

**Scheme 1:**
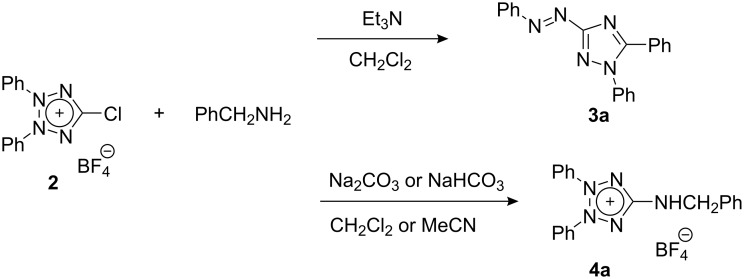
Reaction of chlorotetrazolium **2** with benzylamine.

Next, the reactions of **2** with various amines were examined. When **2** was treated with benzylamine in dichloromethane in the presence of triethylamine, 1,5-diphenyl-3-(phenylazo)-1*H*-1,2,4-triazole (**3a**) was formed in 50% yield. The expected substitution product, 2,3-diphenyl-5-(benzylamino)tetrazolium salt **4a**, was not found in the reaction mixture. Interestingly, when the base was changed to solid sodium carbonate or sodium hydrogencarbonate, **4a** was obtained exclusively (Scheme 1).

The results with other amines are summarized in Table 1. The reaction of butylamine showed a similar tendency: with the triethylamine base triazole **3b** was obtained, whereas with sodium hydrogencarbonate 5-aminotetrazolium **4b** was formed. Other primary amines, such as ethanolamine and ethyl glycinate gave the corresponding triazoles **3c** and **3d**. Interestingly, sterically bulky primary amines as well as secondary amines gave the respective 5-aminotetrazolium salts **4d**–**h**. It is worthy to note that the reaction of isopropylamine/triethylamine gave 3-(isopropylideneamino)-1,5-diphenylformazan as the major product.

**Table 1:** Reaction of chlorotetrazolium **2** with various amines.

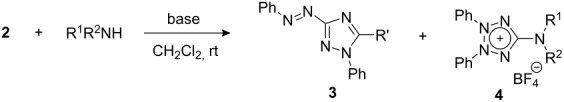							
Entry	R_1_R_2_NH (equiv)		Base (equiv)		Time (h)	Yield (%)	
						**3**	**4**

1^a,b^	PhCH_2_NH_2_	1.0	Et_3_N	3.0	2.5	**3a** 50 (R' = Ph)	0
2^c^	PhCH_2_NH_2_	1.0	NaHCO_3_	1.0	22	0	**4a** 68 (R^1^ = CH_2_Ph, R^2^ = H)
3	PhCH_2_NH_2_	1.0	Na_2_CO_3_	1.0	19	0	**4a** 39
4	*n*-BuNH_2_	1.0	Et_3_N	2.0	72	**3b** 23 (R' = *n*-Pr)	0
5	*n*-BuNH_2_	1.0	NaHCO_3_	1.0	20	0	**4b** 34 (R^1^ = *n*-Bu, R^2^ = H)
6^a,b,c^	HOCH_2_CH_2_NH_2_	1.0	Et_3_N	3.0	3.5	**3c** 50 (R' = CH_2_OH)	**4c** 2 (R^1^ = CH_2_CH_2_OH, R^2^ = H)
7^a,b,c^	NH_2_CH_2_CO_2_Et^d^	1.0	Et_3_N	4.0	4	**3d** 39 (R' = CO_2_Et)	0
8^b,e^	*i*-PrNH_2_	1.0	Et_3_N	1.0	20	0	**4d** 38 (R^1^ = *i*-Pr, R^2^ = H)
9	*i*-PrNH_2_	1.0	NaHCO_3_	1.0	20	0	**4d** 22
10^a,c^	*t*-BuNH_2_	1.4	Et_3_N	1.0	2	0	**4e** 55 (R^1^ = *t*-Bu, R^2^ = H)
11	(PhCH_2_)_2_NH	0.83	Et_3_N	2.0	3	0	**4f** 73 (R^1^ = R^2^ = CH_2_Ph)
12	*n*-Bu_2_NH	1.0	Et_3_N	1.0	4	0	**4g** 96 (R^1^ = R^2^ = *n*-Bu)
13	Et_2_NH	1.0	NaHCO_3_	1.0	18	0	**4h** 41 (R^1^ = R^2^ = Et)

^a^The reaction was conducted under reflux. ^b^Under air. ^c^MeCN was used as a solvent. ^d^The hydrochloride salt was used. ^e^3-(Isopropylideneamino)-1,5-diphenylformazan was obtained (48% yield).

**Scheme 2:**
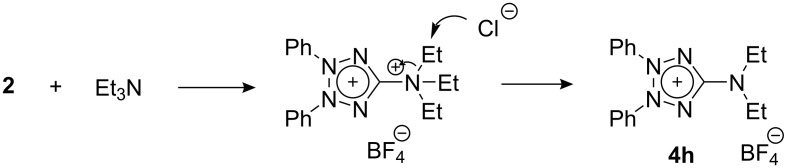
Reaction of chlorotetrazolium **2** with triethylamine.

As a representative tertiary amine, triethylamine was also subjected to the reaction with **2**. The reaction proceeded under reflux in dichloromethane to afford 5-(diethylamino)tetrazolium salt **4h** in 60% yield, presumably via an Arbuzov-type reaction (Scheme 2). The reactions with other nucleophiles than amines such as alcohols and thiols did not give the corresponding oxa- and thiadiazole derivatives.

### Reaction mechanism

**Scheme 3:**
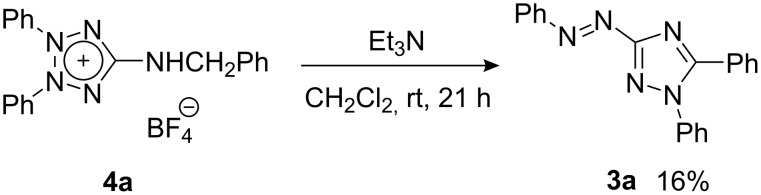
Conversion of benzylaminotetrazolium **4a** to 1,2,4-triazole **3a**.

The reaction mechanism for the formation of triazole **3** was investigated. First, the 5-(benzylamino)tetrazolium salt **4a** was treated with triethylamine in dichloromethane at room temperature. Triazole **3a** was obtained in 16% yield. Although the yield is not high, this fact shows that **3a** is derived, at least partially, from **4a** (Scheme 3).

**Scheme 4:**
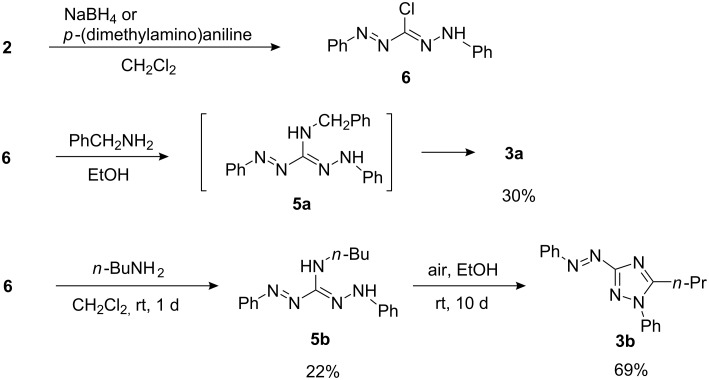
Synthesis of chloroformazan **6** and its reaction with benzylamine and butylamine.

Next, the possibility that **3** is formed by the oxidation of 3-amino-1,5-diphenylformazan **5** was examined. In order to synthesize 3-(benzylamino)formazan **5a**, the reduction of **4a** with reductants such as NaBH_4_ and DIBAL was attempted. However, the desired formazan **5a** was not obtained at all. Then, a nucleophilic substitution of 3-chloroformazan **6** with benzylamine was undertaken. The chlorotetrazolium salt **2** was easily converted to **6** with NaBH_4_ (43% yield) or *p*-(dimethylamino)aniline (73% yield). The reaction of **6** with benzylamine in ethanol, however, did not give the expected **5a**, but triazole **3a** was formed in 30% yield. In this reaction, **3a** is considered to be formed via initially produced formazan **5a**, which is spontaneously oxidized in situ. As these results imply that formazan **5a** is too unstable to be isolated, the preparation of 3-(butylamino)formazan **5b** was next planned. Fortunately, the reaction of **6** with butylamine proceeded and the desired **5b** could be isolated in 22% yield. As expected, aerobic oxidation of **5b** in ethanol gave triazole **3b** in 69% yield, though the reaction took 10 d (Scheme 4).

**Scheme 5:**
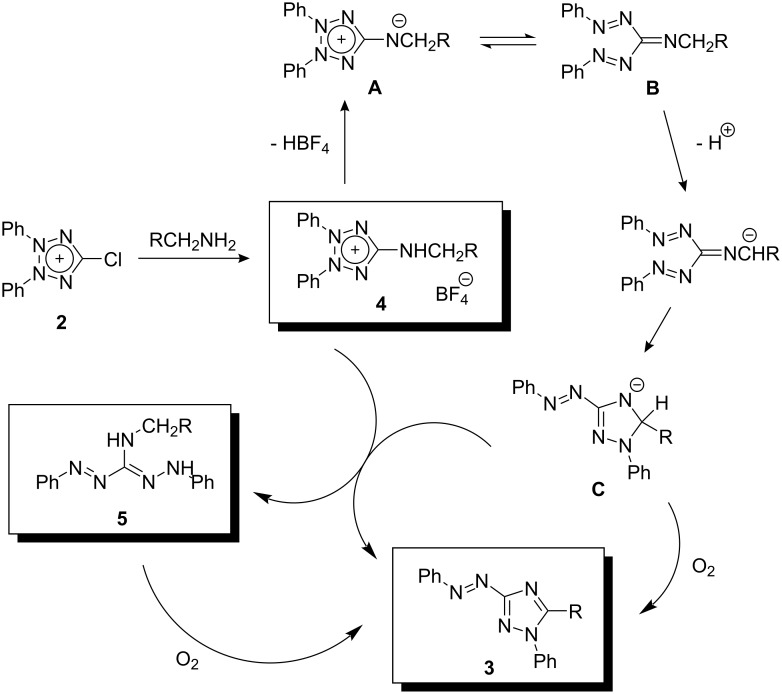
Plausible reaction mechanism for the formation of 1,2,4-triazole **3**.

On the basis of these experimental results, the most plausible reaction mechanism is illustrated in Scheme 5. Nucleophilic substitution of the chlorine atom of **2** by amine gives 5-aminotetrazolium salt **4**. Deprotonation of **4** affords tetazolium-5-aminide **A**, which is in the equilibrium with the acyclic tautomer **B**. Further deprotonation from **B** followed by an intramolecular ring-closure gives **C**, which is oxidized by air to furnish triazole **3**. The sterically demanding primary amines (isopropylamine and *tert*-butylamine) are considered to be difficult to give the cyclized intermediate **C**. The less efficient behaviour of the carbonates in the deprotonation process may be attributed to the heterogeneous reaction system. In the last oxidation stage from **C** to **3**, aminotetrazolium **4** is considered also to act as a hydride acceptor and **4** itself is transformed to 3-aminoformazan **5**. Thus, the formation of triazole **3** is considered to be a dual-path process: via oxidation of the intermediate **C** and formazan **5**. It is worthy to note here that a similar 1,5-dipolar ring closure of type B mesoionic thiocarbonyl ylides is known to furnish thiadiazolines [8].

## Conclusion

It has now been revealed that the 5-chlorotetrazolium salt **2** reacts with primary and secondary amines to give the corresponding 5-aminotetrazolium salts **4**. When triethylamine is employed as a base in the reaction with primary amines, deprotonation from **4** proceeds readily to give tetrazolium-5-aminides **A**. Contrary to the stable type A mesoionic tetrazolium-5-aminides [6–7], type B mesoionic aminide **A** undergoes further transformations from its acyclic tautomer **B** to furnish 1,2,4-triazoles **3**.

## Experimental

### General

Melting points were measured with a hot-stage apparatus Yanaco MP 50533 and are uncorrected. Elemental analyses were carried out with a Perkin Elmer 2400 II CHNS/O. IR spectra were taken as KBr discs on a JASCO A-102 spectrometer. Electronic spectra were measured on a Hitachi U-3500 or a Shimadzu UV-2450 spectrophotometer. ^1^H NMR spectra were obtained using a Varian Mercury 200 (200 MHz) or a Varian Mercury 300 (300 MHz), and ^13^C NMR spectra were obtained using a Varian Mercury 200 (50 MHz). Chemical shifts are recorded in ppm downfield from tetramethylsilane. *J* values are given in Hz. Mass spectra were taken with a Hitachi M-2000 spectrometer (EI, 70 eV). For TLC, Merck Silica gel 60 F_254_ Plate was used. For column chromatography, Merck Silica gel 60 (0.063–0.200 mm) was used.

### 5-Chloro-2,3-diphenyltetrazolium tetrafluoroborate (2)

A mixture of 2,3-diphenyltetrazolium-5-olate (1.0 g, 4.2 mmol) and phosphorus oxychloride (2.2 ml, 24 mmol) was heated at 110 °C for 16 h. The excess phosphorus oxychloride was removed under reduced pressure and the residue was mixed with 42% fluoroboric acid (4.4 ml) and ultrasonicated for 30 min. The mixture was filtered, and the solid was washed with cold THF to give **2** (1.0 g, 72%). From the filtrate and washings, a further amount (0.27 g, 18%) of **2** was obtained; total 1.27 g, 90%. mp 208–212 °C (EtOH); IR (KBr) ν_max_ 3060, 3045, 1482, 1462, 1388, 1362, 1300, 1158, 1122, 1082, 1036, 1000, 788, 780, 764, 692, 684; ^1^H NMR (200 MHz, CD_3_CN) δ 7.67–7.70 (m, 8H), 7.75–7.87 (m, 2H), ^13^C NMR (50 MHz, CD_3_CN) δ 126.29 (*o*-Ph), 131.08 (*m*-Ph), 133.00 (*i*-Ph), 135.14 (*p*-Ph), 158.10 (C^+^); UV/VIS (MeCN): λ_max_ (log ε) = 243.0 (3.41), 272.0 (3.57). Anal. calcd for C_13_H_10_BClF_4_N_4_ (344.5): C, 45.32; H, 2.93; N, 16.26. Found: C, 45.32; H, 3.10; N, 16.52.

### 1,5-Diphenyl-3-(phenylazo)-1*H*-[1,2,4]triazole (3a)

A mixture of **2** (345 mg, 1.0 mmol), benzylamine (110 μl, 1.0 mmol), and triethylamine (0.42 ml, 3.0 mmol) in dichloromethane (6 ml) was heated at reflux for 2.5 h. The reaction mixture was poured into water, and the product was extracted with dichloromethane. The extract was washed with water and diluted hydrochloric acid (1 N). After being dried over Na_2_SO_4_, the solvent was removed and the residue (334 mg) was purified by column chromatography on silica gel (eluent: dichloromethane, then acetonitrile) to give **3a** (163 mg, 50%).

Orange needles, mp 146.7–148.0 °C (EtOH); IR (KBr) ν_max_ 3070, 1594, 1496, 1448, 1380, 1362, 1300, 1178, 1150, 1076, 1020, 990, 924, 916, 800, 770, 740, 718, 690; ^1^H NMR (200 MHz, CDCl_3_) δ 7.35–7.62 (m, 13H), 8.10–8.15 (m, 2H, *o*-PhN=N-); ^13^C NMR (50 MHz, CDCl_3_) δ 123.5, 125.1, 126.8, 128.2, 128.8, 129.0, 129.0, 129.2, 130.3, 132.1, 137.4, 152.4, 155.0, 168.1; MS (EI, 70 eV) *m/z* 325 (M^+^, 3), 220 (4), 180 (100), 117 (24), 77 (53), 31 (13), 27 (54); UV-vis (MeCN) λ_max_ (log ε)/nm: 325 (3.3); Anal. calcd. for C_20_H_15_N_5_ (325.37): C, 73.82; H, 4.65; N, 21.53. Found: C, 73.73; H, 4.44; N, 21.58.

### Reaction of 2 with triethylamine

The salt **2** (175 mg, 0.51 mmol) was treated with triethylamine (140 μl, 1.0 mmol) in dichloromethane (6 ml) under reflux for 16 h. The reaction mixture was poured into water, and the product was extracted with dichloromethane. The extract was washed with diluted hydrochloric acid (1 N). After being dried over Na_2_SO_4_, the solvent was removed to give **4h** (115 mg, 60%).

### Conversion of 4a to 3a

A mixture of **4a** (167 mg, 0.40 mmol) and triethylamine (112 μl, 0.80 mmol) in dichloromethane (4 ml) was stirred at room temperature for 21 h under argon. The reaction mixture was diluted with ether, and washed with diluted hydrochloric acid (1 N) and water. After being dried over Na_2_SO_4_, the solvent was removed and the residue (72 mg) was purified by column chromatography on silica gel (eluent: CH_2_Cl_2_→MeCN) to give a crude product. Recrystallization from ethanol gave **3a** (21 mg, 16%).

### Reduction of chlorotetrazolium salt 2

#### 1. With NaBH_4_

To a solution of **2** (176 mg, 0.51 mmol) in dichloromethane (5 ml) was added NaBH_4_ (7 mg, 0.18 mmol) and the mixture was stirred for 4 h. Additional NaBH_4_ (2 mg, 0.053 mmol) was added and the reaction was continued further for 4 h. Water was added and the product was extracted with dichloromethane. The extract was washed with water and diluted hydrochloric acid (1 N). After being dried over Na_2_SO_4_, the solvent was removed to give **6** (56 mg, 43%).

#### 2. With *p*-(dimethylamino)aniline

A mixture of **2** (345 mg, 1.0 mmol) and *p*-(dimethylamino)aniline (140 mg, 1.0 mmol) in dichloromethane (5 ml) was stirred overnight at room temperature. The solvent was evaporated and the residue was dissolved in acetone. Hexane was added and the resultant black solid was filtered off. Evaporation of the solvent from the filtrate left **6** (190 mg, 73%) as orange crystals.

### Reaction of 3-chloroformazan 6 with benzylamine

A mixture of 3-chloroformazan **6** (130 mg, 0.50 mmol) and benzylamine (0.30 ml, 2.5 mmol) in ethanol (3 ml) was stirred at room temperature for 5 h. The precipitated solid (benzylamine hydrochloride) was filtered off and the filtrate was concentrated. The residue was washed several times with ether. The washings were poured into water and extracted with dichloromethane. The extract was washed with diluted hydrochloric acid (1 N). After being dried over Na_2_SO_4_, the solvent was removed and the residue (141 mg) was purified by column chromatography on silica gel (eluent: CH_2_Cl_2_→MeCN) to give a brown solid (57 mg). This product was further purified by column chromatography on silica gel (eluent: CH_2_Cl_2_) to give triazole **3a** (49 mg, 30%).

### 3-(Butylamino)-1,5-diphenylformazan (5b)

A mixture of **6** (85 mg, 0.33 mmol) and butylamine (100 μl, 1.0 mmol) in dichloromethane (5 ml) was stirred overnight at room temperature. Diluted hydrochloric acid (1 N) was added and the product was extracted with dichloromethane. After being dried over Na_2_SO_4_, the solvent was removed and the residue (80 mg) was purified by column chromatography on silica gel (eluent: CH_2_Cl_2_→MeOH) to give a mixture of *E*/*Z* isomer of **5b** (21 mg, 22%) as a green solid. This compound was easily oxidized by air, and satisfactory elemental analysis data and mass spectrum were not obtained.

^1^H NMR (300 MHz, CDCl_3_) δ 0.90–1.00 (m, 3H), 1.40–1.69 (m, 4H), 3.12 (q), 3.41 (q), 4.90 (br, N*H*), 5.26 (br, N*H*), 6.99 (br, N*H*), 7.15–7.60 (m), 7.78–7.82 (m), 7.89–7.92 (m), 8.08 (s, N*H*), 9.42 (s, N*H*).

^13^C NMR (50 MHz, CDCl_3_) δ 14.0, 14.2, 20.1, 20.5, 31.7, 33.1, 41.7, 43.7, 113.9, 117.9, 121.2, 123.0, 125.3, 128.8, 129.0, 130.6, 143.3, 147.6, 148.0, 151.1, 154.1.

### Oxidation of 5b to triazole 3b

A solution of **5b** (139 mg, 0.47 mmol) in ethanol (20 ml) was allowed to stand at room temperature for 10 d. The solvent was evaporated and the residue was column chromatographed on silica gel (eluent: CH_2_Cl_2_) to give **3b** (94 mg, 69%).

## Supporting Information

File 1 Experimental details for Table 1, entries 2–13
