# Supplementary material for: Synthesis of 3-(phenylazo)-1,2,4-triazoles by a nucleophilic reaction of primary amines with 5-chloro-2,3-diphenyltetrazolium salt via mesoionic 2,3-diphenyltetrazolium-5-aminides
Source: Beilstein J Org Chem. 2009 Mar 2;5:8. doi: 10.3762/bjoc.5.8 (PMC2686317; doi:10.3762/bjoc.5.8)
Supplement: File 1 — Experimental details for Table 1, entries 2–13 [file Beilstein_J_Org_Chem-05-08-s001.doc]

**Supporting Information (Experimental details for Table 1, entries 2**–**13)**

**Synthesis of 3-(phenylazo)-1,2,4-triazoles by a nucleophilic reaction of primary amines with 5-chloro-2,3-diphenyltetrazolium salt via mesoionic 2,3-diphenyltetrazolium-5-aminides**

Shuki Araki*, Satoshi Hirose, Yoshikazu Konishi, Masatoshi Nogura and Tsunehisa Hirashita

Address: Omohi College, Graduate School of Engineering, Nagoya Institute of Technology, Gokiso-cho, Showa-ku, Nagoya 466-8555, Japan

Email: Shuki Araki* - araki.shuki@nitech.ac.jp

* Corresponding author

**Experimental**

**General**

Melting points were measured with a hot-stage apparatus Yanaco MP 50533 and are uncorrected. Elemental analyses were carried with a Perkin Elmer 2400 II CHNS/O. IR spectra were taken as KBr discs on a JASCO A-102 spectrometer. Electronic spectra were measured on a Hitachi U-3500 or a Shimadzu UV-2450 spectrophotometer. ^1^H NMR spectra were obtained using a Varian Mercury 200 (200 MHz) or a Varian Mercury 300 (300 MHz), and ^13^C NMR spectra were obtained using a Varian Mercury 200 (50 MHz). Chemical shifts are recorded in ppm downfield from tetramethylsilane. *J* values are given in Hz. Mass spectra were taken with a Hitachi M-2000 spectrometer (EI, 70 eV). For TLC, Merck Silica gel 60 F_254_ Plate was used. For column chromatography, Merck Silica gel 60 (0.063–0.200 mm) was used.

**5-(Benzylamino)-2,3-diphenyltetrazolium tetrafluoroborate (4a)**

A mixture of **2** (345 mg, 1.0 mmol), sodium bicarbonate (42 mg, 0.50 mmol), and benzylamine (110 μl, 1.0 mmol) in acetonitrile (5 ml) was stirred at room temperature for 22 h under argon atmosphere. The reaction mixture was poured into water, and the product was extracted with dichloromethane. The extract was washed with water and diluted hydrochloric acid (1 N). After being dried over Na_2_SO_4_, the solvent was removed to give **4a** (283 mg, 68%).

Colourless crystals, mp 177.0–181.0 °C (EtOH); IR (KBr) ν_max_ 3200, 3100, 3050, 1630, 1490, 1450, 1360, 1156, 1122, 1082, 1038, 1000, 760, 728, 694. ^1^H NMR (200 MHz, DMSO-*d*_6_) δ 4.62 (d, *J* = 5.8 Hz, 2H, C*H_2_*), 7.31–7.74 (m, 15H, 3Ph), 9.13 (t, *J* = 6.1 Hz, 1H, N*H*). ^13^C NMR (50 MHz, DMSO-*d*_6_) δ 46.2, 126.2, 127.4, 127.6, 128.5, 130.0, 133.0, 133.3, 137.6, 165.2. Anal. calcd. for C_20_H_18_BF_4_N_5_·0.5H_2_O (424.2): C, 56.62; H, 4.51; N, 16.51. Found: C, 56.97; H, 4.20; N, 16.67.

(Table 1, entry 3)

A mixture of **2** (173 mg, 0.50 mmol), sodium carbonate (53 mg, 0.50 mmol), and benzylamine (55 μl, 0.50 mmol) in dichloromethane (4 ml) was stirred at room temperature for 19 h under argon atmosphere. The reaction mixture was filtered and the filtrate was concentrated. The residue (90 mg) was purified by column chromatography on silica gel (eluent: dichloromethane, then acetonitrile) to give **4a** (81 mg, 39%).

**1-Phenyl-3-(phenylazo)-5-propyl-1*H*-[1,2,4]triazole (3b)**

A mixture of **2** (345 mg, 1.0 mmol), butylamine (99 μl, 1.0 mmol), and triethylamine (0.28 ml, 2.0 mmol) in dichloromethane (5 ml) was stirred at room temperature for 72 h under argon atmosphere. The reaction mixture was poured into water, and the product was extracted with ether. The extract was washed with water and diluted hydrochloric acid (1 N). After being dried over Na_2_SO_4_, the solvent was removed and the residue was purified by column chromatography on silica gel (eluent: dichloromethane, then acetonitrile) to give **3b** (66 mg, 23%). An analytical sample was recrystallized from ethanol.

Orange crystals, mp 127.5–128.6 °C (EtOH); IR (KBr) ν_max_ 3070, 2980, 1598, 1500, 1456, 1400, 1370, 1152, 1078, 1056, 1018, 770, 700, 684. ^1^H NMR (200 MHz, CDCl_3_) δ 0.96 (t, *J* = 7.3 Hz, 3H, C*H_3_*), 1.85 (tq, *J* = 7.4 Hz, 2H, C*H_2_*), 2.87 (t, *J* = 7.8 Hz, 2H, C*H_2_*), 7.47–7.54 (m, 8H), 8.06–8.13 (m, 2H, *o*-PhN=N-). ^13^C NMR (50 MHz, CDCl_3_) δ 14.0, 21.5, 28.7, 123.4, 124.9, 128.8, 129.2, 129.3, 131.9, 136.7, 152.4, 157.5, 167.9. MS (EI, 70 eV) *m/z* 291 (M^+^, 100), 248 (7), 214 (41), 186 (31), 146 (100), 117 (100), 105 (98), 104 (79), 103 (5), 91 (98), 78 (49), 77 (100), 76 (10), 65 (71), 51 (97), 43 (33), 28 (42); UV-vis (MeCN) λ_max_ (log ε)/nm: 320 (4.4), 227 (4.2). Anal. calcd. for C_17_H_17_N_5_ (291.4): C, 70.08; H, 5.88; N, 24.04. Found: C, 69.72; H, 5.77; N, 24.08.

**5-(Butylamino)-2,3-diphenyltetrazolium tetrafluoroborate (4b)**

A mixture of **2** (345 mg, 1.0 mmol), sodium bicarbonate (84 mg, 1.0 mmol), and butylamine (99 μl, 1.0 mmol) in dichloromethane (4 ml) was stirred at room temperature for 20 h under argon atmosphere. The reaction mixture was filtered and the filtrate was concentrated. The residue (175 mg) was purified by column chromatography on silica gel (eluent: dichloromethane, then acetonitrile) to give **4b** (130 mg, 34%). An analytical sample was recrystallized from ethanol.

Colourless needles, mp 158.0–158.8 °C (EtOH); IR (KBr) ν_max_ 3160, 3090, 2970, 2875, 1624, 1484, 1458, 1376, 1300, 1144, 1120, 1082, 1040, 996, 766, 730, 692. ^1^H NMR (300 MHz, CD_3_CN) δ 0.99 (t, *J* = 7.5 Hz, 3H, C*H_3_*), 1.47 (sex, *J* = 7.5 Hz, 2H, C*H_2_*), 1.71 (quin, *J* = 7.5 Hz, 2H, C*H_2_*), 3.48 (q, *J* = 6.6 Hz, 2H, NHC*H_2_*), 6.61 (brs, 1H, N*H*), 7.56–7.76 (m, 10H, 2Ph). ^13^C NMR (50 MHz, DMSO-d_6_) δ 13.9, 19.7, 30.8, 42.5, 126.1, 129.9, 133.0, 133.2, 165.1. Anal. calcd. for C_17_H_20_BF_4_N_5_ (381.2): C, 53.56; H, 5.29; N, 18.38. Found: C, 53.21; H, 5.49; N, 18.37.

**5-(Hydroxymethyl)-3-(phenylazo)-1-phenyl-1*H*-[1,2,4]triazole (3c)**

A mixture of **2** (346 mg, 1.0 mmol), ethanolamine (61 μl, 1.0 mmol), and triethylamine (0.42 ml, 3.0 mmol) in acetonitrile (5 ml) was heated at reflux for 3.5 h. The reaction mixture was poured into water, and the product was extracted with dichloromethane. The extract was washed with water and diluted hydrochloric acid (1 N). After being dried over Na_2_SO_4_, the solvent was removed and the residue (219 mg) was purified by column chromatography on silica gel (eluent: CH_2_Cl_2_:MeCN=10:1→MeCN) to give **3c** (146 mg, 50%) and **4c** (7 mg, 2%). An analytical sample of **3c** was recrystallized from ethanol.

Pale orange needles, mp 144.0–148.0 °C (EtOH); IR (KBr) ν_max_ 3250, 3075, 3000, 1618, 1490, 1460, 1360, 1300, 1194, 1152, 1080, 998, 766, 690. ^1^H NMR (200 MHz, CDCl_3_) δ 2.38 (s, 1H, OH), 4.90 (s, 2H, CH_2_), 7.50–7.62 (m, 6H), 7.23–7.78 (m, 2H, *o*-Ph), 8.07–8.11 (m, 2H, *o*-PhN=N-). ^13^C NMR (50 MHz, CDCl_3_) δ 55.5, 123.5, 124.0, 128.9, 129.2, 129.3, 132.4, 136.4, 152.1, 155.7, 167.4. MS (EI, 70 eV) *m/z* 279 (M^+^, 43), 202 (12), 144 (44), 134 (94), 129 (11), 117 (44), 104 (89), 91 (35), 77 (100), 51 (43). UV-vis (MeCN) λ_max_ (log ε)/nm: 319 (4.2), 228 (4.1). Anal. calcd. for C_15_H_13_N_5_O·0.286C_2_H_5_OH (292.466): C, 63.94; H, 5.07; N, 23.95. Found: C, 63.57; H, 4.76; N, 23.87.

**5-(2-Hydroxyethylamino)-2,3-diphenyltetrazolium tetrafluoroborate (4c)**

^1^H NMR (200 MHz, CDCl_3_) δ 1.39 (t, 2H, -C*H_2_*O-), 4.01 (dt, 2H, -NC*H_2_*-), 4.89 (br, 1H, OH), 7.40–7.75 (m, 10H, 2Ph), 8.10 (br, 1H, N*H*).

**5-(Ethoxycarbonyl)-3-(phenylazo)-1-phenyl-1*H*-[1,2,4]triazole (3d)**

A mixture of **2** (346 mg, 1.0 mmol), ethyl glycinate hydrochloride (1.0 mmol), and triethylamine (0.56 ml, 4.0 mmol) in acetonitrile (5 ml) was heated at reflux for 4 h. The reaction mixture was poured into water, and the product was extracted with dichloromethane. The extract was washed with water and diluted hydrochloric acid (1 N). After being dried over Na_2_SO_4_, the solvent was removed and the residue (315 mg) was purified by column chromatography on silica gel (eluent: CH_2_Cl_2_:MeCN=15:1→MeCN) to give **3d** (127 mg, 39%). An analytical sample of **3d** was recrystallized from ethanol.

Brown needles, mp 130.0–132.0 °C (EtOH); IR (KBr) ν_max_ 3060, 3000, 1734, 1596, 1492, 1472, 1436, 1348, 1290, 1222, 1142, 1050, 1014, 920, 844, 762, 696, 680; ^1^H NMR (200 MHz, CDCl_3_) δ 1.36 (t, *J* = 7.2 Hz, 3H, C*H_3_*), 4.41 (q, *J* = 7.2 Hz, 2H, C*H_2_*), 7.52–7.55 (m, 8H), 8.08–8.13 (m, 2H, *o*-Ph); ^13^C NMR (50 MHz, CDCl_3_) δ 14.0, 62.9, 123.5, 125.3, 128.7, 128.9, 129.8, 132.6, 137.1, 145.3, 152.2, 156.7, 167.3. MS (EI, 70 eV) *m/z* 321 (M^+^, 28), 276 (6), 176 (44), 144 (34), 132 (48), 118 (25), 117 (10), 105 (51), 104 (91), 91 (22), 77 (100), 51 (25); UV-vis (MeCN) λ_max_ (log ε)/nm: 315 (4.4), 226 (4.3). Anal. calcd. for C_17_H_15_N_5_O_2_ (321.34): C, 63.54; H, 4.71; N, 21.80. Found: C, 63.35; H, 4.48; N, 21.52.

**5-(Isopropylamino)-2,3-diphenyltetrazolium tetrafluoroborate (4d)**

A mixture of **2** (345 mg, 1.0 mmol), ethylisopropylamine (85 μl, 1.0 mmol), and triethylamine (0.15 ml, 1.0 mmol) in dichloromethane (5 ml) was stirred at room temperature for 20 h. The reaction mixture was poured into water, and the product was extracted with dichloromethane. The extract was washed with water and diluted hydrochloric acid (1 N). After being dried over Na_2_SO_4_, the solvent was removed and the residue (158 mg) was purified by column chromatography on silica gel (eluent: CH_2_Cl_2_→MeOH) to give **4d** (139 mg, 38%).

Orange needles, mp 92.0–97.0 °C (EtOH/hexane); IR (KBr) ν_max_ 3175, 3060, 3090, 1610, 1488, 1460, 1390, 1298, 1176, 1120, 1080, 1040, 996, 762, 730, 684; ^1^H NMR (200 MHz, CDCl_3_) δ 1.36 (d, *J* = 6.4 Hz, 6H, 2C*H_3_*), 4.01 (q, *J* = 6.4 Hz, 1H, C*H*), 5.86 (brs, 1H, N*H*), 7.45–7.67 (m, 10H, 2Ph); ^13^C NMR (50 MHz, CD_3_CN) δ 22.0, 46.7, 126.2, 130.7, 133.5, 134.0, 165.5; UV-vis (MeCN) λ_max_ (log ε)/nm: 258 (sh, 3.7), 236 (sh, 4.0). Anal. calcd. for C_16_H_18_BF_4_N_5_ (367.2): C, 52.34; H, 4.94; N, 19.08. Found: C, 51.85; H, 5.53; N, 17.80.

**3-(Isopropylideneamino)-1,5-diphenylformazan (5c)**

An analytical sample of **4d** was reprecipitated from EtOH/hexane. The water layer of the extraction was neutralized with sodium hydroxide (1N) and extracted with dichloromethane. The extract was dried over Na_2_SO_4_ and the solvent was removed. The residue (176 mg) was purified by column chromatography on silica gel (eluent: CH_2_Cl_2_→MeOH) to give **5c** (134 mg, 48%). An analytical sample of **5c** was recrystallized from EtOH.

Golden needles, mp 158.0–159.0 °C (EtOH); IR (KBr) ν_max_ 3320, 3075, 3000, 2940, 1592, 1548, 1500, 1274, 1200, 1100, 1060, 910, 864, 748, 682; ^1^H NMR (200 MHz, CDCl_3_) δ 1.81 (s, 6H, 2C*H_3_*), 5.19 (s, 1H, N*H*), 6.99–7.06 (m, 1H, Ph), 7.23–7.49 (m, 7H, 2Ph), 7.85–7.89 (m, 2H, Ph); ^13^C NMR (50 MHz, CDCl_3_) δ 28.3, 85.3, 117.0, 122.1, 122.7, 128.8, 128.9, 130.7, 141.5, 152.2, 157.9; MS (EI, 70 eV) *m/z* 279 (M^+^, 32), 264 (85), 174 (15), 134 (8), 133 (7), 118 (20), 105 (22), 93 (7), 92 (69), 91 (8), 77 (100), 65 (17), 51 (16), 32 (22), 28 (98); UV-vis (MeCN) λ_max_ (log ε)/nm: 527 (4.4), 275 (4.1). Anal. calcd. for C_16_H_17_N_5_·0.4C_2_H_5_OH (297.8): C, 67.76; H, 6.57; N, 23.52. Found: C, 68.15; H, 6.25; N, 23.40.

(Table 1, entry 9)

A mixture of **2** (345 mg, 1.0 mmol), sodium bicarbonate (84 mg, 1.0 mmol), and isopropylamine (85 μl, 1.0 mmol) in dichloromethane (4 ml) was stirred at room temperature for 20 h under argon atmosphere. The reaction mixture was filtered and the filtrate was concentrated. The residue (103 mg) was purified by column chromatography on silica gel (eluent: CH_2_Cl_2_→MeCN) to give **4d** (64 mg, 22%). An analytical sample was recrystallized from ethanol.

**5-(*tert*-Butylamino)-2,3-diphenyltetrazolium tetrafluoroborate (4e)**

A mixture of **2** (177 mg, 0.51 mmol), triethylamine (70 μl, 0.50 mmol), and *tert*-butylamine (74 μl, 0.70 mmol) in acetonitrile (4 ml) was refluxed for 2 h under argon atmosphere. The reaction mixture was filtered and the filtrate was washed with water and diluted hydrochloric acid (1 N). After being dried over Na_2_SO_4_, the solvent was removed to give **4e** (107 mg, 55%). An analytical sample of **4e** was recrystallized from ethanol.

Colourless crystals, mp 168.1–172.0 °C (EtOH); IR (KBr) ν_max_ 3150, 3050, 2975, 1602, 1510, 1484, 1460, 1396, 1366, 1296, 1220, 1060, 996, 954, 762, 728, 680. ^1^H NMR (300 MHz, CD_3_CN) δ 1.51 (s, 9H, 3C*H_3_*), 6.60 (br, 1H, N*H*), 7.59–7.74 (m, 10H, 2Ph). ^13^C NMR (50 MHz, CD_3_CN) δ 28.2, 53.3, 126.3, 130.7, 133.6, 134.0, 164.8. Anal. calcd. for C_17_H_20_BF_4_N_5_·0.5C_2_H_5_OH (404.2): C, 53.48; H, 5.74; N, 17.33. Found: C, 53.27; H, 5.55; N, 17.24.

**Reaction of 5-chloro-2,3-diphenyltetrazolium tetrafluoroborate (2) with secondary amines.** **5-(Dibenzylamino)-2,3-diphenyltetrazolium tetrafluoroborate** (**4f**)

A mixture of **2** (413 mg, 1.2 mmol), dibenzylamine (193 μl, 1.0 mmol), and triethylamine (0.33 ml, 2.4 mmol) in dichloromethane (5 ml) were stirred under argon for 3 h. Water was added and the product was extracted with ether. The extracts were washed with water and diluted HCl (1N), and dried over anhydrous Na_2_SO_4_. The solvent was removed and the residue was chromatographed (SiO_2_/CH_2_Cl_2_:MeCN=10:1 to MeCN) to give **4f** (445 mg, 73%). Entry 12 was similarly carried out.

Brass crystals, mp 172–175 °C (EtOH); IR (KBr) ν_max_ 3050, 2925, 1618, 1580, 1486, 1452, 1362, 1336, 1298, 1200, 1156, 1120, 1080, 1032, 996, 942, 768, 724, 694. ^1^H NMR (200 MHz, DMSO-d_6_) δ 4.83 (s, 4H, 2C*H_2_*), 7.33–7.76 (m, 20H, 4Ph). ^13^C NMR (50 MHz, CDCl_3_) δ 51.5, 126.0, 127.5, 127.7, 128.5, 129.6, 132.7, 133.0, 134.6, 166.8. UV-vis (MeCN) λ_max_ (log ε)/nm: 263 (sh, 3.9), 236 (sh, 4.2). Anal. calcd. for C_27_H_24_BF_4_N_5_·0.25H_2_O (509.8): C, 63.60; H, 4.84; N, 13.74%. Found: C, 63.52; H, 4.53; N, 13.72%.

**5-(Dibutylamino)-2,3-diphenyltetrazolium tetrafluoroborate** (**4g**)

Yellow needles, mp 158.2–159.7 °C (EtOH/hexane); IR (KBr) ν_max_ 2960, 2880, 1620, 1488, 1460, 1378, 1334, 1298, 1156, 1120, 1082, 1038, 998, 920, 770, 722, 692; ^1^H NMR (200 MHz, CDCl_3_) δ 0.97 (t, *J* = 7.2 Hz, 6H, 2C*H_3_*), 1.40 (sex, *J* = 7.4 Hz, 4H, 2C*H_2_*), 1.74 (quin, *J* = 7.6 Hz, 4H, 2C*H_2_*), 3.57 (t, *J* = 7.6 Hz, 4H, 2C*H_2_*), 7.45–7.71 (m, 10H, 2Ph); ^13^C NMR (50 MHz, CDCl_3_) δ 13.9, 20.0, 29.0, 49.5, 125.9, 129.7, 132.7, 133.1, 165.8; UV-vis (MeCN) λ_max_ (log ε)/nm: 272 (sh, 3.8), 244 (sh, 4.1). Anal. calcd. for C_21_H_28_BF_4_N_5_ (437.294): C, 57.68; H, 6.45; N, 16.02. Found: C, 57.64; H, 6.74; N, 16.11.

**5-(Diethylamino)-2,3-diphenyltetrazolium tetrafluoroborate** (**4h**)

A mixture of **2** (178 mg, 0.51 mmol), sodium carbonate (42 mg, 0.50 mmol), and diethylamine (52 μl, 0.50 mmol) in dichloromethane (5 ml) was stirred at room temperature for 18 h under argon. The reaction mixture was poured into water, and the product was extracted with dichloromethane. The extract was washed with water and diluted hydrochloric acid (1 N). After being dried over Na_2_SO_4_, the solvent was removed to give **4h** (81 mg, 41%). An analytical sample of **4h** was recrystallized from EtOH.

Yellow crystals, mp 193.4–199.0 °C (EtOH); IR (KBr) ν_max_ 3000, 1624, 1496, 1462, 1160, 1086, 1040, 1000, 780, 700; ^1^H NMR (200 MHz, CDCl_3_) δ 1.35 (t, *J* = 7.0 Hz, 6H, 2C*H_3_*), 3.66 (q, *J* = 7.0 Hz, 4H, 2C*H_2_*), 7.46–7.69 (m, 2Ph); ^13^C NMR (50 MHz, CDCl_3_) δ 12.5, 43.8, 126.0, 130.0, 132.8, 133.1, 165.3. Anal. calcd. for C_17_H_20_BF_4_N_5_·0.5C_2_H_5_OH (404.2): C, 53.48; H, 5.74; N, 17.33. Found: C, 53.64; H, 5.37; N, 17.03.
